# Disrupting vaccine logistics

**DOI:** 10.1093/inthealth/ihab010

**Published:** 2021-03-11

**Authors:** Eric R James

**Affiliations:** Sanaria Inc, 9800 Medical Center Drive, Rockville, MD 20850, USA

**Keywords:** cold chain, distribution, logistics, vaccines

When the Pfizer–BioNTech coronavirus disease 2019 (COVID-19) vaccine BNT162b2 was first authorized for emergency use in December 2020, it became, after the Ebola virus vaccine Ervebo, only the second human vaccine requiring distribution at between −80°C and −60°C. In the weeks leading up to this authorization, the specification for storage and distribution using ultra-low-temperature freezers and dry ice was being portrayed by the media as a potential ‘logistical nightmare’.^1^ Adopting this cold chain means a new packaging paradigm: vials stored in freezers are transferred into trays of 195 five-dose vials (six doses if low dead space syringe–needle combinations are used), with up to five trays per carton, packed under 23 kg of dry ice in a ‘thermal shipper’ insulated box. And in a departure from common procedures for distribution, Pfizer organized and controlled the shipping operation, contracting third-party logistics (3PL) companies to transport vaccine to distribution hubs with newly installed ultra-low-temperature freezers and subsequent onward transport directly to the immunization sites. Distribution to clinics has appeared to work reasonably well (organization and coordination of administration at the clinic level has been another story). Concerns over the temperature used for storage and shipping this vaccine appear now to be subsiding.

Other coronavirus vaccines are stored and transported at −20°C (e.g., mRNA 1273 from Moderna, the first iteration of the Russian Sputnik V vaccine) or between 2°C and 8°C (most others including the AstraZeneca–Oxford University AZD1222 adenovirus vectored vaccine and JNJ-78436735 from Johnson & Johnson). The conventional assumption, and not just by the media, was that the ease of distributing vaccines is directly related to the temperature of the cold chain: low- and middle-income countries (LMICs) generally do not have networks of ultra-low-temperature freezers. However, Pfizer believes that its cold chain can be extended into LMICs and has pledged to supply 50 million doses to Africa.^2^ Pfizer's vaccine was the first, and currently still the only, COVID-19 vaccine to receive World Health Organization (WHO) emergency use authorization with distribution through COVID-19 Vaccines Global Access. This will change, as expectations are that the AstraZeneca–Oxford and J&J vaccines may eventually lead the pack for several reasons (cheaper, perhaps single dose, more rapid manufacture), including their 2–8°C cold chain requirement.

## Changing the cold standard

The 2–8°C cold chain has been the basic industry standard for vaccine storage and transportation and is used for all current US Food and Drug Administration (FDA)-licensed human vaccines, with the exception of smallpox, chickenpox, shingles and one of the measles, mumps, and rubella II vaccines that are distributed between −25°C and −15°C and Ervebo (for Ebola virus). Immunization programs in LMICs are built around 2–8°C distribution; however, distribution systems at other temperatures can and do operate effectively in these countries. The target populations for most vaccines in LMICs are children served by the Expanded Programme on Immunization (EPI). This 2–8°C distribution is also the common standard for vaccine cold chains in high-income countries and has become the default: any vaccine that does not fit this mould is considered problematic, sometimes irrationally so, thus the initial negative portrayal in the media^1^ of Pfizer's logistics operation despite a vaccine efficacy of 95%.

The WHO stipulates that a ‘vaccine or any component presented for prequalification should not require storage at less than −20°C’.^3^ Furthermore, if a vaccine must be stored ‘below +2°C during its shelf-life period, it should have a minimum period of storage above +2°C of 6 months’. Ultra-low-temperature (−80°C) and cryogenic (< −150°C) cold chains do not fit into these traditional recommendations for acceptance of new vaccines. Nevertheless, Ervebo upended this stipulation, setting a new precedent and also becoming the fastest vaccine yet to move through the approval and prequalification process.

Ervebo is currently the sole licensed (by the FDA, European Medicines Agency, Ghana, Burundi, Zambia and the Democratic Republic of the Congo [DRC]), WHO-prequalified human vaccine that is distributed between −80°C and −60°C. Ervebo was initially successfully distributed under compassionate use protocols to aid in containing the Ebola outbreaks in West Africa in 2014–2016^4^ and more recently in the DRC,^5^ demonstrating that a vaccine with ultra-low temperature requirements can be deployed in LMIC settings when the need is especially critical. The Ervebo cold chain bears little resemblance to a standard 2–8°C cold chain and its creation stimulated significant innovation and the introduction of new equipment: for the first leg, vaccine vials were shipped to the main distribution hubs in insulated boxes with dry ice; from there to satellite stores at −60°C in Arktek shipping units using pre-conditioned phase-change panels to maintain temperature for up to 6 d; and finally the vaccine was thawed, loaded into syringes and 12 syringes sent same day in Cryo-Q cold boxes to the immunization clinics.^4^

## Cold chain extremes

The holy grail for vaccine developers and logisticians is a vaccine that can be stored and transported at ambient temperatures. No licensed vaccine yet meets this standard, although Vivotif, the orally administered vaccine against typhoid fever, formulated as enteric-coated capsules, is probably closest. Several candidate vaccines in early-stage development employ ‘new’ methods of thermostabilization such as spray drying and foam drying^6^ that may yet yield licensed products stable at ambient temperatures. At the other end of the scale, the chimeric antigen receptor T-cell (CAR-T) products Kymriah and Yescarta and the malaria vaccine Sanaria®PfSPZ Vaccine, now entering phase 3 trials, contain cryopreserved live eukaryotic cells that are stabilized by cryopreservation and are stored and transported at < −150°C using liquid nitrogen vapour phase (LNVP) as the refrigerant.

## Limits of the chains

There are advantages and disadvantages to storage and distribution with each of the four categories of temperature-defined cold chains (2–8°C, −20°C, −80°C and < −150°C). The main disadvantage of the 2–8°C cold chain is its reliance on refrigerators that can only maintain the temperature for short periods when electricity supply is interrupted—a not infrequent scenario in LMICs. Propane or solar-powered refrigerators can provide independence from the electricity grid or from generators. Nevertheless, even with a reliable electricity supply, as in high-income countries, maintaining vaccine within the narrow window of 2–8°C is not easy and temperature excursions are common. Perhaps counterintuitively, freezing may be the most frequent cause of vaccine loss during transportation and storage,^7^ with vaccines that contain alum as adjuvant being particularly sensitive. Both freezing and high-temperature excursions reduce vaccine potency, so to protect vaccines, vial monitoring methods such as labels with thermosensitive components that change colour based on the extent and duration of out-of-specification temperatures are used to report thermal histories. CAR-T products are shipped with real-time monitors recording temperature, humidity, tilt, shock and Global Positioning System location data for each shipment; some of these parameters are monitored for the Pfizer and Moderna vaccine shipments. Increasingly, many 3PL and courier companies are including electronic monitoring devices, accessible via mobile device apps, for distribution of high-value products.

**Figure 1.**
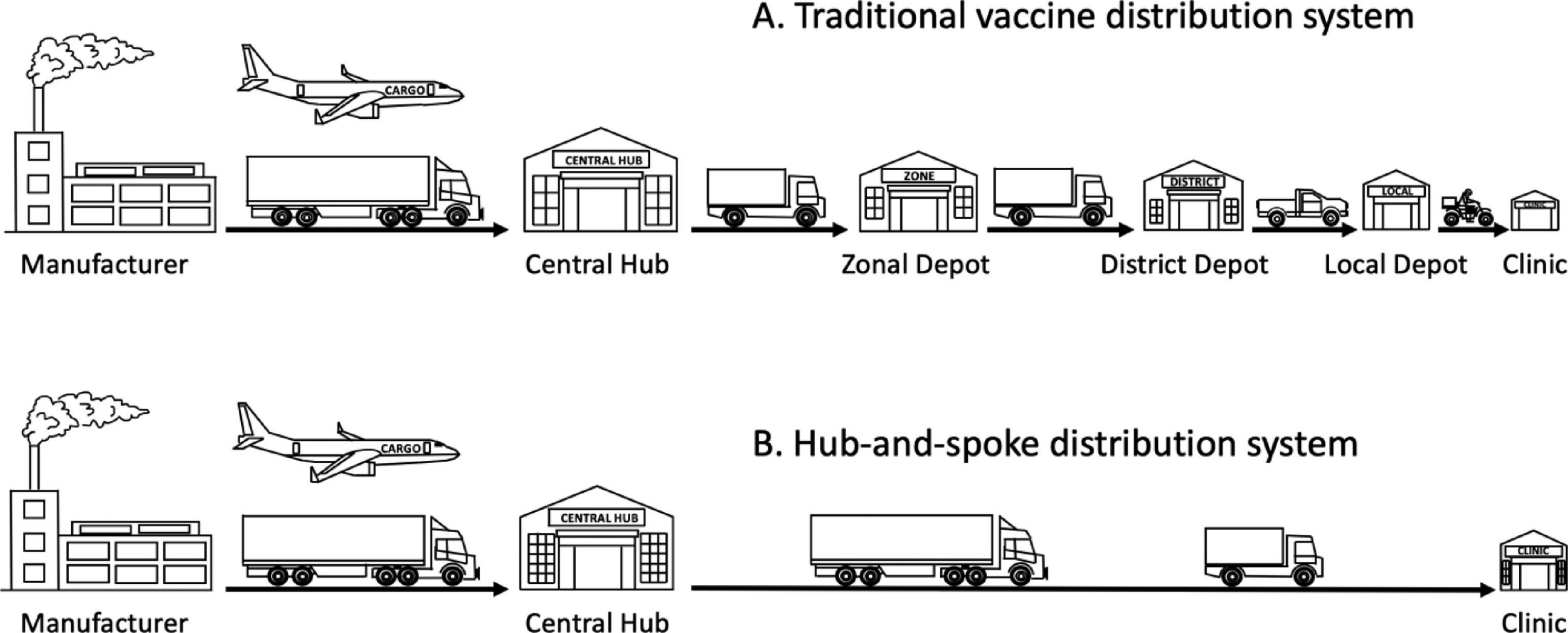
Comparison between the traditional vaccine distribution system, similar to that of the EPI, and the hub-and-spoke system, similar to that used in distributing childhood vaccines for the Centers for Disease Control.

A second reason why typical 2–8°C and −20°C cold chains can be problematic is their design. The standard EPI cold chain utilizes a sequence of storage facilities extending from the manufacturer to the immunization clinic that rely on refrigerators and freezers. For example, the vaccine cold chain in Tanzania, established by the Medical Stores Department and now run by the EPI,^8^ consists of the main hub in Dar es Salaam, nine zonal depots, up to 24 district depots in each zone and finally a total of 8,497 (in 2020) health facilities (hospitals, health centres, clinics and dispensaries). Such multistop distribution networks (Figure 1) provide multiple opportunities for temperature excursions, handling errors and equipment failure. (The Tanzanian cold chain is actually better than many and has been one of the most studied). Simplified distribution networks with three or even two stops—a central depot supplying immunization clinics directly—improve control and reduce the chance for temperature and equipment failure. This hub-and-spoke model of distribution (Figure 1) is used in the USA for distributing childhood vaccines for the Centers for Disease Control directly from two central hubs, as well as seasonal influenza vaccines and now the Moderna COVID-19 vaccine in partnership with the US government's Warp Speed program. Similar simplified networks for the EPI have been trialled successfully in recent years in Senegal (the Moving Warehouse study of Project Optimize)^9^ and in Tunisia and Vietnam. LNVP-based < −150°C cold chains utilize the hub-and-spoke model.

## Vaccine containers

All vaccines, except those distributed at cryogenic temperatures, are manufactured in glass vials closed using a synthetic stopper held in place by a crimped aluminum cap. The main drawback to this vial design is that it limits storage temperatures to > −80°C. However, product stability improves as the temperature of storage decreases, down to the glass transition temperature (approximately −135°C for aqueous-based products). For vaccines stored at 2–8°C, a one-log loss in potency over 12–18 months is quite common and accepted in the industry. In marked contrast, vaccines stored at cryogenic temperatures < −150°C experience no loss in potency over many years of storage. With glass vials, the seal between the vial and its stopper opens at temperatures < −80°C due to the differential contractility of the components and loss in elasticity of the stopper. To maintain container closure integrity for products stored at cryogenic temperatures, different containers are employed. CAR-T products, where volumes are larger, use bags or plastic vials accessed by sealed tubes (e.g. Cook Regentec, Indianapolis, IN, USA). High-throughput filling, cryogenic storage and distribution of vaccines in smaller volumes can be accomplished with closed-fill vials (Aseptic Technologies, Gembloux, Belgium) or with plastic vials containing a seal-protected septum (SiO_2_ Materials Science, Auburn, AL, vials). By using such containers, the benefits of the cryogenic cold chain option could be extended to other vaccines.

## Advantages of LNVP

Cold chains using LNVP are also more efficient and easier to operate than those using ultra-low-temperature freezers and dry ice, which is especially difficult to obtain in many LMICs. Liquid nitrogen is available almost everywhere in the world, and LNVP cryoshippers, after arriving in the clinic, double as temporary storage repositories for 7–14 d, or longer with liquid nitrogen recharges, and need no electricity. There are around 11 veterinary vaccines, including the cattle vaccine against East Coast Fever in East Africa, that are transported using liquid nitrogen as refrigerant and the LNVP cold chain supports a huge international livestock breeding industry. PfSPZ Vaccine against malaria has been successfully distributed to >13 clinical trials sites, including to 9 sites in 6 countries of sub-Saharan Africa, using the LNVP-based cold chain. For reasons of controllability and efficiency, certain types of refrigerator trucks equipped with piping injection systems use liquid nitrogen for transporting vaccines and other products not only at < −150°C, but also at −20°C and −80°C.

## Not just the vaccine

Ancillary supplies accompanying all vaccines include syringes, needles, disinfecting wipes and safety boxes for sharps. Additionally, several frozen vaccines, those that come in multidose vials (e.g. Pfizer's COVID-19 vaccine) and vaccines that are lyophilized also require a diluent. These components are shipped at ambient temperatures, but their cumulative bulk and weight can be significant. For 1 million doses of the Pfizer–BioNTech vaccine, >205 thermal shippers cumulatively weighing ∼7.5 metric tons including dry ice and occupying 19 m^3^ are required. And the ancillary supplies include >1.2 million syringes with needles and 200 000 vials (containing a total of 400 L) of saline diluent. The estimated weight and volume of these ancillary supplies is ∼16 metric tons occupying >250 m^3^. Cumulatively, this amounts to a transportation capacity requirement equivalent to three fully loaded 18-wheeler articulated trucks with 16 m trailers, and this must be repeated again for the second dose of a two-dose regimen vaccine.

## Legacy disruption

The speed with which the new COVID-19 vaccines have been developed has been spectacular. Complementing this has been the ramp up in distribution logistics. At the main vaccine distribution hubs in the USA, Europe and elsewhere for the Pfizer and Moderna vaccines and others, logistics companies, largely with funding from governments, have invested heavily in the infrastructure to rapidly handle tens of millions of doses. This infrastructure includes the ultra-low-temperature freezer farms (warehouses) that have featured prominently in the media, and experience is continuing to improve the last-mile distribution to the immunization sites. One legacy of sudden acute respiratory syndrome coronavirus 2 will be multiple improvements to vaccine distribution systems at all cold chain temperatures, but particularly a tried and tested ultra-low-temperature cold chain capable of handling vaccines at scale against the next pandemic and, in the meantime, available to encourage development of variants of existing vaccines to take advantage of the −80°C to −60°C cold chain distribution infrastructure.
